# Bone marrow-derived exosomes promote inflammation and osteoclast differentiation in high-turnover renal osteodystrophy

**DOI:** 10.1080/0886022X.2023.2264396

**Published:** 2023-10-23

**Authors:** Rao Fu, Kexin Meng, Rui Zhang, Xuanyi Du, Jundong Jiao

**Affiliations:** a Department of Nephrology, The Second Affiliated Hospital of Harbin Medical University, Harbin, China; b Institute of Nephrology, Harbin Medical University, Harbin, China

**Keywords:** Exosomes, renal osteodystrophy, osteoclast, inflammation, miRNA

## Abstract

**Introduction:**

Renal osteodystrophy (ROD) is a type of bone metabolic disorder in patients with chronic kidney disease (CKD). Inflammation is associated with bone loss in ROD. However, its precise mechanism has not yet been elucidated. The present study was conducted to investigate whether exosomes (Exos) in bone marrow (BM) are involved in the pathogenesis of high-turnover ROD.

**Methods:**

Bone mass, osteoclast number, and pro-inflammatory cytokines levels of BM supernatant were detected in adenine-induced ROD rats. The effect of Exos derived from BM (BM-Exos) of ROD (ROD-Exos) on inflammatory genes and osteoclast differentiation of BM-derived macrophages (BMMs) were further examined. Then, exosomal miRNA sequencing was performed and an miRNA-mRNA-pathway network was constructed.

**Results:**

we found increased osteoclasts and decreased bone mass in ROD rats, as well as inflammatory activation in the BM niche. Furthermore, BMMs from ROD rats displayed overproduction of proinflammatory cytokines and increased osteoclast differentiation, accompanied by nuclear factor κB (NF-κB) signaling activation. Mechanistically, we found that ROD-Exos activates NF-κB signaling to promote the release of proinflammatory cytokines and increase osteoclast differentiation of BMMs. Meanwhile, a total of 24 differentially expressed miRNAs were identified between BM-Exos from ROD and normal control (NC). The miRNA-mRNA-pathway network suggests that rno-miR-9a-5p, rno-miR-133a-3p, rno-miR-30c-5p, rno-miR-206-3p, and rno-miR-17-5p might play pivotal roles in inflammation and osteoclast differentiation. Additionally, we validated that the expression of miR-9a-5p is upregulated in ROD-Exos.

**Conclusion:**

The BM niche of ROD alters the miRNA cargo of BM-Exos to promote inflammation and osteoclast differentiation of BMMs, at least partially contributing to the pathogenesis of high-turnover ROD.

## 1. Introduction

Renal osteodystrophy (ROD), an important complication of chronic kidney disease (CKD), is responsible for the high incidence of fractures [1,2] and an increased rate of hospitalization in CKD patients [3]. ROD is a metabolic bone disorder characterized by abnormal bone remodeling [4], accompanied by a pattern of changes in serum calcium, phosphate, parathyroid hormone (PTH), vitamin D, and fibroblast growth factor 23 (FGF-23) [5]. In CKD patients with higher parathyroid function, the bone cells are hyperactivated, leading to high turnover ROD. In those patients, osteoclastic bone resorption is accompanied by pathological osteoblastic bone formation and mineralization, which is associated with cortical bone thinning and osteoporosis. Although recent clinical studies have demonstrated the association of inflammation with low bone mass and mineral metabolism abnormalities among CKD patients [6,7], the underlying mechanisms have not yet been completely elucidated.

Exosomes (Exos) are small (30–150 nm in diameter) membrane vesicles that are secreted into extracellular microenvironments by most cell types. Exos may transfer various molecules, including proteins, mRNA/miRNA, and DNA, to adjacent or distant cells and thus play a crucial role in the regulation of cell-cell communication [8,9]. Increasing evidence indicates that Exos can indirectly promote osteoclast differentiation by proinflammatory cytokines or directly by transported cargo. For example, Marton et al. showed that blood-derived Exos could regulate human osteoclastogenesis in distinct inflammatory arthropathies [10]. Moreover, the contents of Exos, specifically miRNAs, change with the local microenvironment as well as different disease states, such as in the synovial fluid of patients with osteoarthritis [11]. We therefore hypothesized that the bone marrow (BM) niche of ROD alters BM-derived Exos (BM-Exos) to promote the production of proinflammatory cytokines and osteoclast differentiation of BM-derived macrophages (BMMs).

In the present study, we first identified elevated levels of proinflammatory cytokines in the BM niche of ROD, suggesting the role of inflammation in the pathogenesis of ROD. We further found that BM-Exos from ROD rats (ROD-Exos) could be taken up by BMMs, leading to the overproduction of proinflammatory cytokines, increased osteoclast differentiation, and activated nuclear factor κB (NF-κB) signaling. Finally, we uncovered the changes in the miRNA profiles of ROD-Exos by using miRNA sequencing, analyzed the relationships among differentially expressed miRNAs, target mRNAs, and inflammation- and osteoclast differentiation-related signaling pathways, and validated the upregulation of miR-9a-5p in ROD-Exos. We hope our results will help us understand the role of BM-Exos in high-turnover ROD pathogenesis.

## 2. Materials and methods

### 2.1. Animal procedures

Male Sprague–Dawley rats at 8 weeks of age were received from the Animal Center of The Second Affiliated Hospital of Harbin Medical University. All experimental procedures were approved by the Institutional Animal Care and Use Committee of The Second Affiliated Hospital of Harbin Medical University. The ROD rat model was established as previously described [12]. Rats were fed a diet containing 1.11% calcium, 1.03% phosphorus, 9% protein, and 1000 IU/kg vitamin D3 (modified AIN-93 standard diet; Keao Xieli Feed Co., Ltd, Beijing, China). After 1 week of acclimatization, the rats were randomly assigned into two groups: 15 rats were used to establish an ROD model group, which switched to an adenine-enriched diet (to add 0.75% adenine to modified AIN-93 standard diet) for 4 weeks following the standard diet for 2 weeks; 12 rats were included in the normal control (NC) group, which were fed the standard diet throughout the study. For dynamic parameter evaluation of bone histomorphometry, rats were injected intraperitoneally with 30 mg/kg alizarin red (Solarbio, Beijing, China), a fluorescent bone marker, at 13, 12, 3, and 2 days before sacrifice. After 6 weeks, rats were anesthetized and sacrificed by exsanguination *via* abdominal aortic puncture.

### 2.2. Serum biochemical assays

Serum creatinine, blood urea nitrogen (BUN), calcium, and phosphate were measured by colorimetric assay kits (Elabscience). Serum PTH was tested by an enzyme-linked immunosorbent assay (ELISA) kit (Elabscience).

### 2.3. Bone histomorphometry

The left proximal tibia of each rat was removed and fixed in 100% ethanol. After dehydration, samples were embedded in methyl methacrylate (MMA), cut into sections using a Thermo HM360 microtome (Thermo Fisher, USA) and stained with a Goldner or TRAP staining kit (Solarbio, Beijing, China). Histomorphometric parameters were measured by ImageJ analysis software. All parameters were evaluated based on standardized nomenclature and formulae recommended by the American Society of Bone and Mineral Research (ASBMR) [13].

### 2.4. Micro-computed tomography (micro-CT) bone imaging

Femurs were imaged by micro-CT (SCANCO, Switzerland). Briefly, samples were fixed in the scanning tube, the scanning range was determined, then many continuous images could be obtained by scanning along the long axis of bone, images of interesting region were reconstructed to obtain three-dimensional structure of trabecular bone and cortical bone.

### 2.5. BMM isolation and osteoclast differentiation

Bone marrow cells were harvested from the femurs and tibias of Sprague–Dawley rats. The cell suspension was centrifuged at 1000 r/min for 5 min, and pellets were resuspended in 3 mL erythrocyte lysate for 1 min. Then, 5 mL α-MEM was added and centrifuged at 1000 r/min for 5 min. The cells were cultured overnight in α-MEM supplemented with 10% fetal bovine serum (FBS, ScienCell, USA) and 100 U/mL penicillin/streptomycin (Gibco, USA) in 100 mm tissue culture dishes. Nonadherent cells were harvested, and cultured in 30 ng/ml macrophage colony-stimulating factor (M-CSF; PeproTech, USA), and adherent cells were used as BMMs.

To induce osteoclast differentiation, BMMs (1 × 10^5^/mL) were seeded in 24-well plates and cultured in the presence of 30 ng/mL M-CSF and 50 ng/mL receptor activator of nuclear factor-κB ligand (RANKL; PeproTech, USA) for 4–6 days.

### 2.6. Tartrate-resistant acid phosphatase (TRAP) staining

To identify osteoclasts, cells were fixed and stained with a TRAP staining kit according to the manufacturer’s instructions. TRAP-positive cells with three or more nuclei were considered osteoclasts and counted under a light microscope (Zeiss, Germany).

### 2.7. ELISA

Supernatants were collected from BMMs cultured for 24 h or BM mixed with phosphate-buffered saline (PBS) (1:10) by centrifugation (1000 × g for 20 min). The levels of the proinflammatory cytokines interleukin-1β (IL-1β), interleukin-6 (IL-6), and tumor necrosis factor-α (TNF-α) in BMMs and BM supernatant were determined by ELISA kits (Elabscience).

### 2.8. RNA extraction and quantitative real-time PCR (qRT–PCR) assay

Total RNA was extracted using TRIzol reagent (Invitrogen) according to the manufacturer’s protocol. Complementary DNA (cDNA) was generated from 500 ng RNA using a TransScript cDNA synthesis kit (Transgen, Beijing, China). qRT–PCR was performed with FastStart Universal SYBR® Green Master Mix (Roche, Germany). Relative gene expression was calculated using the 2^-ΔΔCt^ method normalized to GAPDH (internal control). The primers were synthesized by Thermo Fisher Scientific and are listed in Table 1.

**Table 1.** Primers used for qRT–PCR.

Marks	Forward/reverse	Sequence	Gene accession no
TNF-α	Forward	5-ATG GGC TCC CTC TCA TCA GT-3′	NM_012675.3
	Reverse	5′-GCT TGG TGG TTT GCT ACG AC-3′	
IL-1β	Forward	5′-GTC CTC TGC CAA GTC AGG TC-3′	NM_031512.2
	Reverse	5′-CAG GGA GGG AAA CAC ACG TT-3′	
IL-6	Forward	5′-CCC ACC AGG AAC GAA AGT CA-3′	NM_012589.2
	Reverse	5′-ACT GGC TGG AAG TCT CTT GC-3′	
NF-κB_1_	Forward	5′-CTG AGT CCC GCC CCT TCT AA-3′	NM_001276711.1
	Reverse	5′-CTC CAC CAG CTC TTT GAT GGT-3′	
I-κB	Forward	5′-TGA GTA CCT GGA CTT GCA GAACG-3′	NM_030867.2
	Reverse	5′-TGT AGA TGC CTC TCC AAG GATGG-3′	
MMP-9	Forward	5′-GAT CCC CAG AGC GTT ACT CG-3′	NM_031055.2
	Reverse	5′-GTT GTG GAA ACT CAC ACG CC-3′	
CTSK	Forward	5′-TCC TCA ACA GTG CAA GCG AA-3′	NM_031560.2
	Reverse	5′-CCA GCG TCT ATC AGC ACAGA-3′	
GAPDH	Forward	5′-AGT GCC AGC CTC GTC TCA TA-3′	NM_017008.4
	Reverse	5′-ACC AGC TTC CCA TTC TCA GC-3′	

### 2.9. BM-Exos isolation and characterization

Exos were isolated from BM using Total Exosome Isolation Reagent (Life Technologies, USA). Briefly, BM separated from the rat femur or tibia was resuspended in 500 μl PBS, and the supernatant was collected by centrifugation at 2000× g for 30 min to remove cells and debris. Supernatant and reagent (100 µl: 20 µl) were mixed and incubated at 4 °C for 30 min followed by centrifugation at 10,000 × g for 10 min. Deposited exosome particles were completely resuspended in 200 μl of PBS. For exosome-specific markers, TSG101 and CD9 were detected by Western blot (WB). For transmission electron microscopy (TEM), exosome suspensions were dropped onto copper grids and negatively stained with 20 g/L phosphotungstic acid. Then, images were obtained using TEM (HT7700, Japan). The particle size was determined by the dynamic light scattering (DLS) method, and reports were generated automatically by a Zetasizer Nano ZS (Malvern Instruments, UK).

### 2.10. Uptake of BM-Exos by BMMs

Exos isolated from NC and ROD rat BMs were labeled with PKH26 (Sigma–Aldrich) and incubated with BMMs (1 × 10^5^/mL) for 12 h. After incubation, cells were fixed with 4% formaldehyde for 30 min and mounted with DAPI mounting media (Beyotime). Images were taken with a confocal microscope (Zeiss, Germany).

### 2.11. Exosome treatment

Normal BMMs (1 × 10^6^/mL) were cultured with 10% exosome-depleted media and treated with PBS, NC-Exos (20 µg/mL) or ROD-Exos (20 µg/mL). After 36 h of treatment, the gene expression levels of IL-1β, IL-6, TNF-α, NF-κB_1,_ and IκB were tested using qRT–PCR. Osteoclast differentiation assays were conducted as mentioned above in the presence of M-CSF (30 ng/mL) and RANKL (50 ng/mL) for 3 days followed by qRT–PCR of osteoclast-specific genes (MMP-9 and CTSK) and 6 days followed by TRAP staining.

### 2.12. miRNA sequencing and bioinformatics analysis

miRNAs in both NC-Exos and ROD-Exos (*n* = 5) were sequenced using the Illumina HiSeq2500 platform. After quality control, the clean reads were aligned to the reference genome through Bowtie software. The mapped reads were compared with the mature miRNA sequences in the known miRNA database (miRBase V22), and aligned reads were identified as known miRNAs. TPM was used to calculate the expression of miRNAs, and |log2(FC)|> =0.584962500721156 and P value< =0.05 were the thresholds for identifying differentially expressed miRNAs. MultiMiR was used to predict the target mRNAs of miRNAs. Functional enrichment analysis of target mRNAs of differentially expressed miRNAs was performed using Gene Ontology (GO) and Kyoto Encyclopedia of Genes and Genomes (KEGG).

### 2.13. miRNA isolation and qRT–PCR assay

miRNAs were isolated from BM-Exos using miRNeasy Kit (Qiagen, USA) according to manufacturer’s protocol. The concentration of miRNA was determined using a NanoDrop spectrophotometer (Thermo Scientific, USA). cDNA was generated from 200 ng miRNAs using miScript II RT Kit (Qiagen, USA). qRT–PCR was performed with SYBR Green I and miR specific primers (Qiagen, USA). RNU6 (RNA, U6 small nuclear 2) and SNORD (small nucleolar RNA, C/D box) were used as normalization control genes for miRNA expression. Relative expression of miRNA was evaluated by using 2^-ΔΔCt^ method.

### 2.14. Statistical analysis

Data are presented as the mean ± standard deviation (SD). The statistical significance between groups was measured using Student’s *t* test or one-way ANOVA followed by Bonferroni posttest. Data analyses were performed using SPSS 24.0 and GraphPad Prism 8.0. A *p* value < 0.05 was considered statistically significant.

## 3. Results

### 3.1. Increased osteoclasts and bone loss in adenine-induced ROD model

**Figure 1.**
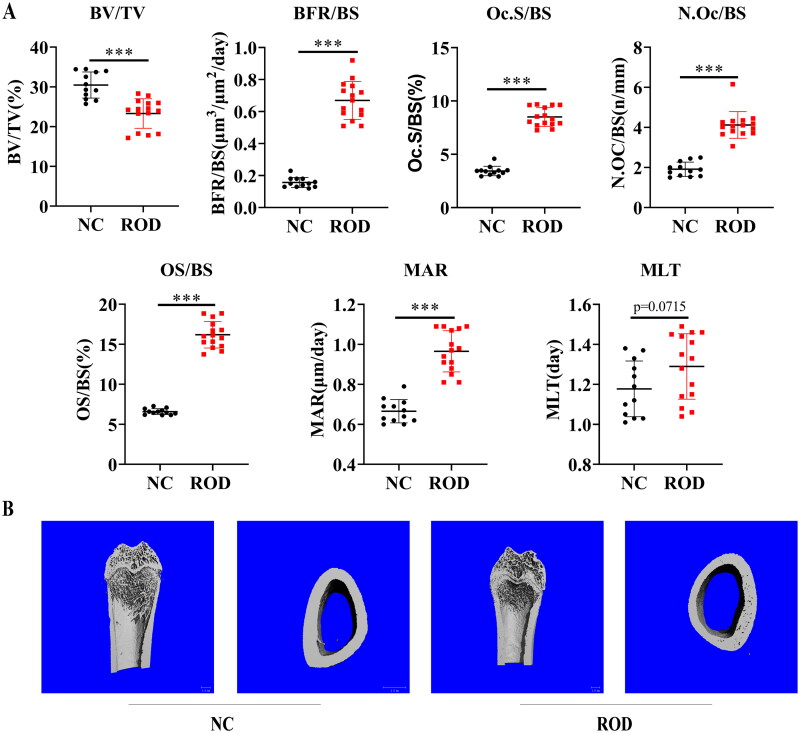
Increased osteoclasts and bone loss in adenine-induced ROD model. **(A)** Bone histomorphometry data. BV/TV, bone volume/tissue volume; BFR/BS, bone formation rate; Oc.S/BS, osteoclast surface per millimeter bone perimeter; N.Oc/BS, osteoclast number per millimeter bone perimeter; OS/BS, osteoid surface/bone surface; MAR, mineral apposition rate; MLT, mineralization lag time. *n* = 12-15 per group, ***p < 0.001 or as indicated, student’s t-test. **(B)** Representative micro-computed tomography images of distal femurs of NC and ROD rats.

We first established a high-turnover ROD model in rats by feeding an adenine and high phosphorus diet as previously described [12]. Adenine administration leads to abnormal kidney function and abnormal mineral metabolism with high serum phosphorus, high PTH, and low serum calcium (Table 2). Bone histomorphometric analysis revealed static and dynamic bone parameters are disturbed in ROD rats (Figure 1A), which included decreased bone volume (ROD: 23.3%±3.7% versus NC: 30.1%±3.3%), increased bone formation rate (ROD: 0.67 ± 0.12 μm3/μm2/day versus NC: 0.16 ± 0.03 μm3/μm2/day), increased osteoclast surface (ROD: 8.5%±0.9% versus NC: 3.4%±0.45) and number (ROD: 4.1 ± 0.7/mm versus NC: 1.9 ± 0.4/mm), increased osteoid (ROD: 16.2%±1.7% versus NC: 6.65 ± 0.4%), increased mineral apposition rate (ROD: 0.97 ± 0.10 μm/day versus NC: 0.67 ± 0.06 μm/day), but mineralization lag time was no significant differences between two groups (ROD: 1.3 ± 0.2 day versus NC: 1.2 ± 0.1 day). We also found erosion pores of different sizes in the cross-section of cortical bone and decreased trabecular number in ROD rats by micro-CT (Figure 1B). The above findings indicate that the ROD rats developed bone loss and increased osteoclasts.

**Table 2.** Serum biochemical parameters of rats.

Parameters	NC (*n* = 12)	ROD (*n* = 15)
BUN (mmol/L)	7.77 ± 1.35	13.63 ± 3.09***
Cr (μmol/L)	57.32 ± 12.99	178.37 ± 25.27***
Ca (mmol/L)	2.55 ± 0.21	2.25 ± 0.20**
P (mmol/L)	2.30 ± 0.22	3.48 ± 0.51***
PTH (pg /mL)	54.63 ± 21.00	1025.40 ± 201.16***

Data represent the mean ± SD. NC: normal control group; ROD: renal osteodystrophy group; BUN: blood urea nitrogen; Cr: creatinine; Ca, calcium; P phosphate; PTH: parathyroid hormone. **p < 0.01, ***p < 0.001.

### 3.2. Elevated proinflammatory cytokines in BM niche and BMMs of ROD rats

**Figure 2.**
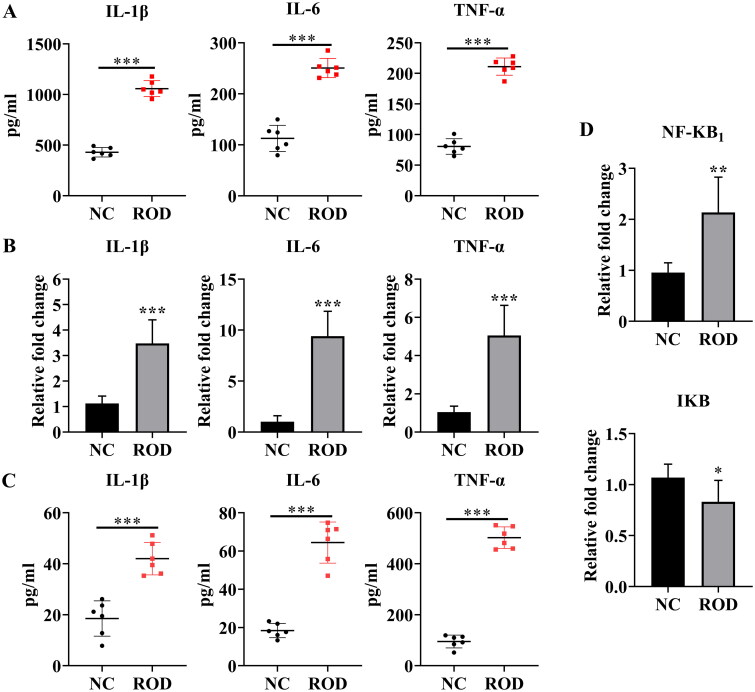
Increased proinflammatory cytokines in BM supernatant and BMMs of ROD rats. **(A)** ELISA was used to detect the concentrations of IL-1β, IL-6, and TNF-α in the BM supernatant of rats. **(B)** BMMs from NC or ROD rats were cultured for 24 h followed by RT–PCR of IL-1β, IL-6, and TNF-α. **(C)** ELISA was used to detect the concentrations of IL-1β, IL-6, and TNF-α in the BMM supernatants of rats in the two groups. **(D)** BMMs from NC or ROD rats were cultured for 24 h followed by RT–PCR of NF-κB_1_ and IκB. Data represent the mean ± SD, *n* = 6 per group, ***p < 0.001, **p < 0.01, *p < 0.05.

Considerable evidence suggests that chronic inflammation is related to local and systemic bone loss [14]. To reveal the role of inflammation in the pathogenesis of ROD, we investigated whether inflammation exists in the BM niche (origin of osteoclasts) of ROD rats. As shown in Figure 2A, compared to the NC group, the levels of IL-1β, IL-6, and TNF-α in the BM supernatant of the ROD group increased markedly (*p* < 0.001), suggesting the activation of an inflammatory status in the BM niche of ROD rats.

Macrophages are the main producers of inflammatory cytokines [15]. Therefore, we further studied whether the production of proinflammatory cytokines was increased in BMMs of ROD rats. To test this hypothesis, the gene expression of IL-1β, IL-6, and TNF-α was detected. As shown in Figure 2B, IL-1β increased threefold (*p* < 0.001), and TNF-α increased fivefold (*p* < 0.001), whereas IL-6 showed a dramatic increase with a tenfold upregulation (*p* < 0.001) in BMMs from ROD rats, compared to NC group. Consistent with these results, the concentrations of IL-1β, IL-6, and TNF-α in the BMM supernatant of ROD rats were also significantly elevated, as indicated in Figure 2C. In parallel, we found that NF-κB signaling genes were differentially expressed between the two groups. Compared to the control group, NF-κB_1_/p50 gene expression increased (*p* < 0.01), whereas IκB decreased (*p* < 0.05) in the ROD group (Figure 2D). These results suggest that the synthesis and secretion of proinflammatory cytokines and the activity of NF-κB signaling were increased in BMMs.

### 3.3. ROD-Exos promote the production of proinflammatory cytokines in BMMs

**Figure 3.**
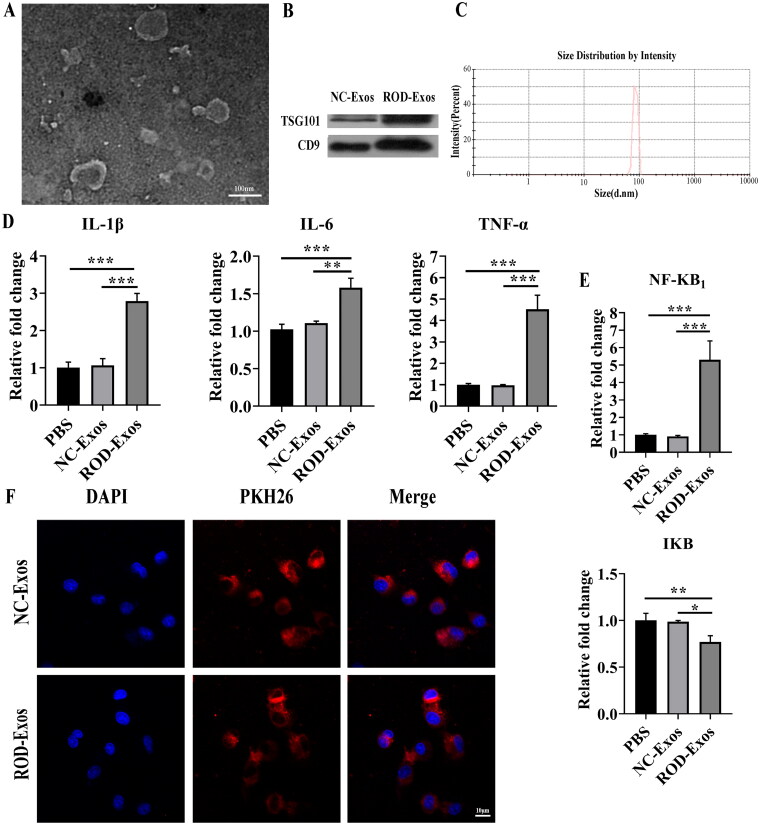
Effect of ROD-Exos on the proinflammatory activity of BMMs. **(A)** Transmission electron microscope images of Exos. **(B)** Western blot for exosome-associated proteins TSG101 and CD9. **(C)** The size distribution of Exos was measured by dynamic light scattering. **(D, E)** BMMs were treated with PBS, NC-Exos or ROD-Exos for 36 h followed by qRT–PCR of IL-1β, IL-6, TNF-α, NF-κB_1_, and IκB. Data represent the mean ± SD, *n* = 3, ***p < 0.001, **p < 0.01, *p < 0.05. **(F)** PKH26-labeled BM-Exos were cultured with BMMs for 12 h, and uptake was observed using laser confocal microscopy. Blue, DAPI-stained nuclei; red, PKH26-labeled Exos.

Recent studies suggest that Exos are involved in regulating inflammatory responses [16]. Thus, we further examined whether BM-Exos were involved in the pathogenesis of ROD by regulating the inflammation of BMMs. Exos were isolated from the BM of NC and ROD rats using precipitation and centrifugation methods and characterized using TEM, WB and DLS. We found that BM-Exos were spherical with a bilayer membrane structure under TEM (Figure 3A). WB analysis showed bands of exosome-specific markers TSG101 and CD9 (Figure 3B). The size of BM-Exos was in the ∼100 nm diameter range by DLS (Figure 3C).

Then, we determined whether ROD-Exos could promote the production of proinflammatory cytokines in BMMs. Normal BMMs were treated with PBS, NC-Exos, or ROD-Exos. Compared with the other two treatment groups, administration of ROD-Exos significantly enhanced the proinflammatory activity of BMMs, as evidenced by upregulated gene expression of IL-1β, IL-6, and TNF-α (Figure 3D). To further explore the proinflammatory mechanisms of ROD-Exos, we tested the effects of ROD-Exos on inflammatory signaling. As seen in Figure 3E, ROD-Exos significantly upregulated the gene expression of NF-κB_1_ but downregulated the gene expression of IκB, suggesting that ROD-Exos might promote the production of proinflammatory cytokines *via* NF-κB signaling. Additionally, an Exos uptake experiment was performed. As shown in Figure 3F, PKH26 red fluorescently labeled Exos were observed inside the BMMs, located in the perinuclear and cytoplasmic regions, suggesting that these BM-Exos could function by being taken up by BMMs.

### 3.4. ROD-Exos promote osteoclast differentiation of BMMs

**Figure 4.**
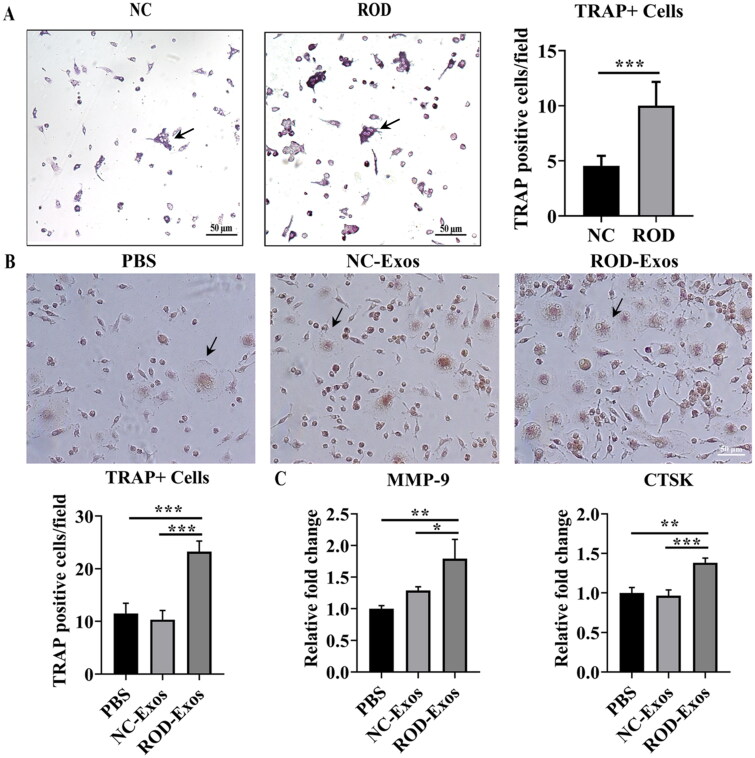
Effect of ROD-Exos on osteoclast differentiation of BMMs. **(A)** Primary BMMs (1 × 10^5^/mL) were cultured with M-CSF and RANKL for 4 days followed by TRAP staining. TRAP^+^ cells with three or more nuclei (black arrows in panel A) were defined as osteoclasts. Magnification: 100×. **(B)** Normal primary BMMs (1 × 10^6^/mL) were cultured with M-CSF and RANKL and treated with PBS, NC-Exos or ROD-Exos for 6 days followed by TRAP staining. The number of TRAP^+^ cells with three or more nuclei (black arrows in panel B) was counted. Magnification: 200×. **(C)** BMMs were cultured with M-CSF and RANKL and treated with PBS, NC-Exos or ROD-Exos for 3 days followed by qRT–PCR of osteoclast-specific genes (MMP-9 and CTSK). Data represent the mean ± SD. *n* = 6 per group **(A)**; *n* = 3 per group **(B, C)**. ***p < 0.001,**p < 0.01, *p < 0.05.

Previous studies reported that inflammation plays a role in promoting osteoclast differentiation. Because ROD-Exos enhance the proinflammatory activity of BMMs, we investigated whether the osteoclast differentiation ability is concomitantly increased by ROD-Exos. We conducted an osteoclast differentiation assay for 4 days followed by TRAP staining and found that osteoclast differentiation of ROD-derived BMMs was enhanced significantly compared to that of the NC group (Figure 4A). Next, we explored whether the increased osteoclast differentiation ability of BMMs was mediated by ROD-Exos. The results showed that treatment with ROD-Exos led to a significant increase in osteoclast number (Figure 4B) and upregulation of MMP-9 and CTSK gene expression (Figure 4C) in BMMs. This finding suggests that ROD-Exos play a positive role in the osteoclast differentiation of BMMs.

### 3.5. Changes of BM-Exo miRNA expression in ROD

**Figure 5.**
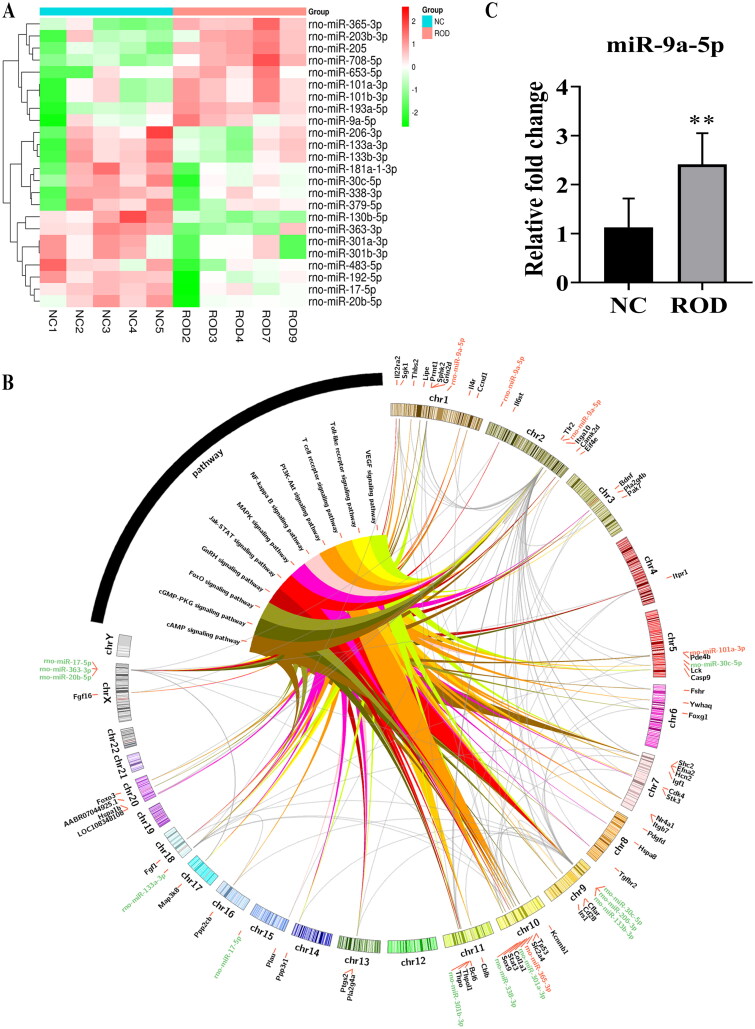
Profiles for differentially expressed miRNAs. **(A)** Heatmap of differentially expressed miRNAs between NC-Exos and ROD-Exos. *n* = 5. **(B)** The circos plot showing the miRNA-mRNA-pathway network. Red represents upregulated miRNAs, green represents downregulated miRNAs, and black represents target mRNAs. **(C)** qRT–PCR showing upregulation of miR-9a-5p expression in ROD-Exos. Data represent the mean ± SD, *n* = 6, **p < 0.01.

Emerging evidence shows that exosomal miRNAs are involved in regulating inflammation and bone remodeling [17,18]. Therefore, we detected differentially expressed miRNAs between NC-Exos and ROD-Exos using miRNA sequencing. A total of 24 differentially expressed miRNAs (9 upregulated and 15 downregulated miRNAs) were obtained in ROD-Exos compared with NC-Exos, as shown in Figure 5A. Based on the results of enrichment analysis, we constructed a miRNA-mRNA-pathway network (Figure 5B), showing that a total of 60 target mRNAs of 13 differentially expressed miRNAs were enriched in 11 inflammation- and osteoclast differentiation-related signaling pathways. We found that rno-miR-9a-5p, rno-miR-133a-3p, rno-miR-30c-5p, rno-miR-206-3p, and rno-miR-17-5p could act on a wide range of targets, making them pivotal in inflammation and osteoclast differentiation. In addition, NF-κB signaling, which is changed in ROD-derived BMMs and normal BMMs treated with ROD-Exos, was also included in the results of enrichment analysis.

### 3.6. Upregulated miR-9a-5p expression in ROD-Exos

To validate the reliability of the sequencing results, miR-9a-5p was selected for qRT–PCR and the result showed significant (*p* < 0.01) upregulation of miR-9a-5p in ROD-Exos compare to NC-Exos (Figure 5C). Meanwhile, according to the results of enrichment analysis (Supplementary Table1), we found miR-9a-5p could act on NF-κB_1_ and thus participate in the regulation of inflammation- and osteoclast differentiation-related signaling pathways. These results indicated that Exos miR-9a-5p may be involved in the pathogenesis of high-turnover ROD, but further mechanism studies are still needed.

## 4. Discussion

ROD is a type of bone disorder characterized by impaired bone quality and strength and increased bone pain, bone deformity, and fracture risk in CKD patients [19]. Studies showed that high-turnover bone disease is the most classic and prevalent type of ROD in predialysis CKD patients [20,21]. Rapid cortical bone loss occurs with elevated bone turnover in these patients [22]. However, the precise mechanisms of bone loss in CKD patients with ROD have not yet been completely elucidated. Here, we established a high-turnover ROD model and found bone loss in both trabecular and cortical bones, as well as a greater number of osteoclasts and resorption lacunae. Consistent with the increased number of osteoclasts *in vivo*, osteoclast differentiation of ROD-derived BMMs increased significantly compared to the normal control group.

The role of inflammation in promoting osteoclast differentiation and bone loss has received extensive attention [23]. Recently, Lin et al. demonstrated that proinflammatory cytokines could activate NF-κB signaling in macrophages to promote osteoclast differentiation and bone loss [24]. PTH amplifies its role in promoting osteoclast differentiation and bone resorption by increasing TNF-α production [25]. In patients with end-stage renal disease, proinflammatory cytokines (IL-6 and TNF-α) are also attributed to osteoporosis [6]. CKD is considered a persistent microinflammatory state [26], which has been well established by serology [27,28] and renal histopathology [29]. While levels of cytokines in the bone marrow environment are more stable than in the blood and may give a better account of their effect on bone condition [30]. In addition, bone loss appears to be more strongly associated with the inflammatory BM niche than systemic inflammation because osteoclasts originate from hematopoietic stem cells (HSCs) in BM. In the present study, we first identified elevated levels of proinflammatory cytokines in the BM niche of ROD. Concomitantly, ROD-derived BMMs were shown to overproduce and release proinflammatory cytokines, accompanied by NF-κB signaling activation, which are closely associated with the BM inflammation and increased osteoclast differentiation ability in ROD. Thus, CKD may create a proinflammatory BM microenvironment that contributes to progressive bone loss.

Exos are membrane vesicles (30–150 nm in diameter) released from most cell types, which circulate in various body fluids, such as blood, urine, synovial fluid, ascites, and BM interstitial fluid, and play a crucial role in intercellular communications [8,31]. Recent studies support that Exos may be involved in the progression of inflammatory diseases. Essandoh et al. demonstrated that Exos isolated from LPS-treated RAW264.7 cells could promote the production of TNF-α and IL-6 in macrophages [32]. Bretz et al. indicated that Exos from amniotic fluid or ovarian cancer ascites stimulated the release of proinflammatory cytokines from monocytes and mouse BMMs *via* NF-κB signaling [33]. Consistently, we demonstrated that ROD-Exos could upregulate the gene expression of proinflammatory cytokines and activate NF-κB signaling in BMMs. To further verify the proinflammatory effects of ROD-Exos, we performed an Exo uptake experiment and confirmed that PKH26-labeled BM-Exos could be internalized by BMMs to regulate their function. Singleton et al. showed that extracellular vesicles (EVs) derived from the BM of traumatic brain injury mice increase osteoclast differentiation of bone marrow cells by NF-κB signaling [34]. Likewise, our results show that ROD-Exos can promote osteoclast differentiation and upregulate the expression of osteoclast-specific genes (MMP-9 and CTSK) in BMMs.

Exos can deliver pro-/anti-inflammatory contents, including proteins and RNAs, to recipient cells to participate in regulating inflammatory responses [35]. Davis et al. reported that Exos are abundant within BM interstitial fluid and that the miRNA cargo changes with the different local microenvironments [36]. In our study, miRNA sequencing of NC-Exos and ROD-Exos suggests that this is indeed the case. We found that 13 differentially expressed miRNAs may be involved in inflammation and osteoclast differentiation of BMMs. This result is consistent with previous studies. Zhang et al. demonstrated that miR-9-5p could target SIRT1 and promote particle-induced osteoclastogenesis *via* the NF-kB signaling pathway [37]. Najm et al. reported that miR-17 reduced the number of osteoclasts and the production of proinflammatory cytokines by targeting JAK1 and STAT3, thus playing an anti-inflammatory and anti-erosive role in rheumatoid arthritis [38]. A recent paper by Jin et al. revealed that miRNA-30c-5p could inhibit inflammation by the eIF2α/ATF4 pathway [39]. Therefore, we speculate that these differentially expressed miRNAs carried by BM-Exos participate in regulating the production of proinflammatory cytokines in BMMs and are associated with bone loss in high-turnover ROD.

Our current study has validated that the expression of miR-9a-5p is upregulated in ROD-Exos. The results of enrichment analysis showed that miR-9a-5p could act on a wide range of targets including NF-κB1 and thus participate in the regulation of inflammation- and osteoclast differentiation-related signaling pathways. Based on these results and our findings that ROD-Exos promote the release of proinflammatory cytokines and increase osteoclast differentiation of BMMs, we speculate that elevated level of miR-9a-5p in ROD-Exos might play important role in the pathogenesis of high-turnover ROD. Further studies are needed to demonstrate direct relationship between Exos miR-9a-5p cargo and NF-κB signaling activation/osteoclast differentiation.

A limitation of our current study is that we did not conduct in-depth functional verification of differentially expressed miRNAs. Further studies are needed to focus on the targets of differentially expressed miRNAs in BMMs to clarify its role in the pathogenesis of high-turnover ROD. Another significant exploration in the future will be to investigate whether the role of BM-Exos in animal models is consistent with our *in vitro* experiments.

This is the first study, to our knowledge, to identify the elevated levels of proinflammatory cytokines in the BM niche of ROD and demonstrate miRNA profiling in BM-Exos of ROD. Our data indicate that the BM niche of ROD alters the miRNA cargo of BM-Exos to promote inflammation and osteoclast differentiation of BMMs, contributing to the bone loss of ROD.

## Supplementary Material

Supplemental Material

## Data Availability

All data generated or analyzed during this study are included in this article. Further inquiries can be directed to the corresponding author.
